# The Multifaceted Effects of Non-Steroidal and Non-Opioid Anti-Inflammatory and Analgesic Drugs on Platelets: Current Knowledge, Limitations, and Future Perspectives

**DOI:** 10.3390/ph17050627

**Published:** 2024-05-14

**Authors:** Alexandros Tsoupras, Despina A. Gkika, Ilias Siadimas, Ioannis Christodoulopoulos, Pavlos Efthymiopoulos, George Z. Kyzas

**Affiliations:** Hephaestus Laboratory, Department of Chemistry, School of Science, Democritus University of Thrace, GR 65404 Kavala, Greece; dgkika@chem.ihu.gr (D.A.G.); pefthymi@chem.ihu.gr (P.E.); kyzas@chem.duth.gr (G.Z.K.)

**Keywords:** antiplatelet, NSAIDs, drugs, ibuprofen, ketoprofen, diclofenac, naproxen, paracetamol, platelet, platelet aggregation

## Abstract

Non-steroidal anti-inflammatory drugs (NSAIDs) are among the most widely utilized pharmaceuticals worldwide. Besides their recognized anti-inflammatory effects, these drugs exhibit various other pleiotropic effects in several cells, including platelets. Within this article, the multifaceted properties of NSAIDs on platelet functions, activation and viability, as well as their interaction(s) with established antiplatelet medications, by hindering several platelet agonists’ pathways and receptors, are thoroughly reviewed. The efficacy and safety of NSAIDs as adjunctive therapies for conditions involving inflammation and platelet activation are also discussed. Emphasis is given to the antiplatelet potential of commonly administered NSAIDs medications, such as ibuprofen, diclofenac, naproxen and ketoprofen, alongside non-opioid analgesic and antipyretic medications like paracetamol. This article delves into their mechanisms of action against different pathways of platelet activation, aggregation and overall platelet functions, highlighting additional health-promoting properties of these anti-inflammatory and analgesic agents, without neglecting the induced by these drugs’ side-effects on platelets’ functionality and thrombocytopenia. Environmental issues emerging from the ever-increased subscription of these drugs are also discussed, along with the need for novel water treatment methodologies for their appropriate elimination from water and wastewater samples. Despite being efficiently eliminated during wastewater treatment processes on occasion, NSAIDs remain prevalent and are found at significant concentrations in water bodies that receive effluents from wastewater treatment plants (WWTPs), since there is no one-size-fits-all solution for removing all contaminants from wastewater, depending on the specific characteristics of the wastewater. Several novel methods have been studied, with adsorption being proposed as a cost-effective and environmentally friendly method for wastewater purification from such drugs. This article also presents limitations and future prospects regarding the observed antiplatelet effects of NSAIDs, as well as the potential of novel derivatives of these compounds, with benefits in other important platelet functions.

## 1. Introduction

Platelets, the smallest blood constituents, play a central role in thrombosis and hemostasis, critical processes for presventing hemorrhage due to vessel damage. Hemostasis encompasses the processes following an initial platelet activation that result in platelet accumulation and subsequent activation of blood coagulation pathways, which are recognized as the primary platelet-mediated mechanisms for generating thrombin [1]. The subsequent platelet adhesion and aggregation play a crucial role in the formation of a platelet plug, essential for halting bleeding and achieving hemostasis. The coagulation and inflammatory pathways work in tandem as vital components of the host defense system, with complementary functions aimed at limiting tissue damage, combating pathogens, and restoring physiological balance through the regulation of these mechanisms [2]. Apart from the important roles of platelets in thrombosis and hemostasis, platelet activation also plays a significant role in inflammatory responses, which however if not resolved can lead to several platelet-activation-related thrombo-inflammatory manifestations linked to various inflammation-associated disorders, such as atherosclerosis and cardiovascular diseases (CVD), neurodegenerative and renal disorders, allergies, diabetes, and cancer, among others [3,4,5].

Platelet activation is triggered by the interaction of released thrombo-inflammatory mediators that act as platelet agonists with specific platelet surface receptors. Platelets are activated by inflammatory mediators released from other cells, which act agonistically on platelet surface receptors and/or platelet activated due to several reasons can also release thrombo-inflammatory mediators that can further activate platelets and other cells and thus can also serve as the initial trigger of inflammatory responses. The agonist–receptor complexes on platelet surfaces play a critical role in this platelet activation and aggregation process and in initiating subsequently several other thrombo-inflammatory manifestations, including the coagulation cascade [1]. Therefore, although platelets were initially thought their main role is to prevent blood loss following tissue injury, extensive research has shown that they also play crucial roles in the progression of pathogenic processes associated with acute vascular atherothrombotic diseases and cancer [6].

Several natural or synthetic compounds, which can inhibit platelet activation and aggregation by affecting each or more of these pathways, have been proposed, not only for antiplatelet pharmaceuticals but also as antithrombotic and anti-inflammatory cardio-protective, anti-tumor and neuroprotective agents [4,5]. Even though anti-platelet therapy is crucial for the prevention and treatment of several disorders in which platelets are implicated, and especially for CVD prevention and treatment [6,7], it still raises the risk of upper gastrointestinal tract side effects and bleeding. Available antiplatelet drugs interfere with one or more steps in platelet release and aggregation [8] processes, leading to a measurable reduction in thrombosis risk but also an increased risk of bleeding [9]. The impact of all antiplatelet drugs on aggregation has been clinically researched extensively [10,11]. Nevertheless, not only classic antiplatelet drugs can serve as compounds for reducing platelet activation and aggregation. Since inflammation is tightly associated and triggered or is triggered by platelet activation, several compounds with anti-inflammatory potential have also been studied for potential antiplatelet effects [12]. They represent some of the world’s most widely used prescription and over-the-counter medications, while some of them like ibuprofen and aspirin have been listed by the World Health Organization (WHO) in the list of essential medicines.

NSAIDs are critically important pain medications, with aspirin and ibuprofen, listed. Early pharmacological studies identified key differences in the potency of NSAIDs between the two isoforms of COXs, COX-1, and COX-2. COX-2 selective NSAIDs are comparable with traditional non-selective drugs for treating pain and inflammation with reduced gastrointestinal side effects. These drugs also have the potential to prevent and treat cancer and some members of the class including ibuprofen can produce anti-platelet effects [13]. For some NSAIDs, despite their widespread use and therapeutic benefits, even short-term use of less than a week has been linked with an elevated risk of thrombotic cardiovascular events. Thus, now there is a major concern across all forms of NSAID therapy for the cardiovascular side effects associated with these drugs, which were only realized in the post-2000s era. On the other hand, the co-prescription of NSAIDs with antithrombotics in patients with CVD has been linked to a significant independent risk of bleeding [14].

NSAIDs exhibit diverse structural and pharmacodynamic characteristics, albeit sharing similar modes of action [15], and their classification spans various chemical categories [16]. Extensive research has been conducted on drug interactions between NSAIDs and antithrombotic medications due to their potential for serious consequences. Concurrent use of NSAIDs and antithrombotic drugs is linked to an elevated risk of bleeding, particularly gastrointestinal bleeding [17]. However, concomitant NSAID use is associated with increased risks of both bleeding and thromboembolism [18].

Pharmacokinetic data for NSAIDs are also crucial in therapeutic decision-making, particularly by aiding in decisions that prioritize both safety and efficacy. It should be noted that no single NSAID demonstrates significantly superior efficacy, so assessing safety profiles becomes paramount before making therapeutic choices. This assessment should carefully consider the nature of the pain requiring treatment and individual patient characteristics. When comparing NSAIDs, disparities in pharmacokinetics and pharmacodynamics are readily apparent. In the absence of prospective randomized studies, drug selection can be informed by these parameters, while also considering risks associated with drug-drug interactions, advanced age, organ impairment, dosage, formulation, protein binding, therapeutic half-life, and other clinically relevant pharmacological attributes [19].

This study addresses the call made by Driver et al. [20] by shedding light on the insufficiently studied impact of paracetamol (acetaminophen) on platelets. Furthermore, it builds upon the assertions of Abrignani et al., who suggested that among NSAIDs, aceclofenac, diclofenac, and ibuprofen pose lower bleeding risks when prescribed alongside antiplatelet agents compared to lornoxicam, piroxicam, and desketoprofen/ketoprofen [21]. At present, the scientific literature lacks comprehensive answers to several significant issues regarding the selection of non-steroidal and non-opioid anti-inflammatory and analgesic drugs concerning their impact on platelets, particularly in inhibiting various pathways of platelet activation. The following unresolved issues are briefly outlined: Firstly, although pharmaco-epidemiological studies and meta-analyses of randomized controlled trials indicate variations in the risk associated with different NSAIDs, there exists substantial heterogeneity across studies in estimating comparative risk. Secondly, while traditional NSAIDs are classified into several chemical categories, it remains uncertain which NSAID from each representative group holds the highest potency.

In light of these unresolved issues, we conducted an analysis investigating six NSAIDs and meticulously selected individual NSAIDs representing all chemical groups. We examined their potential to interfere with platelet function inhibition both in vivo and In vitro. Additionally, we investigated the effects of paracetamol in this context. Thus, the aim of this review is fourfold: (i) to elucidate the capacity of NSAIDs to influence platelet function by inhibiting various pathways of platelet activation, (ii) to clarify the impact of each NSAID on platelet function and compare the antiplatelet actions and cardio-protective effects of aspirin when co-administered with NSAIDs, (iii) to determine the most potent NSAID within each group and assess its risk/benefit potential, and (iv) to shed light in limitations and future perspectives for the pleiotropic bio-functionality of these drugs on platelets and bring in surface the lack of data from the so far contacted research on the efficacy of these drugs on all platelet reactivity pathways (i.e., against the platelet-activating factor pathway).

## 2. Methods

During the process of selecting the digital libraries for the automated search strategy, we chose to utilize the widely recognized Scopus database for the following reasons: (i) its extensive coverage of research across various scientific fields and (ii) the availability of robust tools for systematic searches [22,23]. The final search query comprised the following terms: “(diclofenac, naproxen OR ibuprofen, ketoprofen, paracetamol (acetaminophen)): AND platelet, AND antiplatelet, AND antithrombotic, AND Collagen, AND ADP, AND PAF, AND Thrombin, AND Arachidonic acid, AND Epinephrine, AND eicosanoids, AND prostaglandins, AND leukotrienes”. This query was applied to the titles, abstracts, and keywords of articles, and the search process was concluded in April 2024.

### 2.1. Inclusion Criteria

The selection criteria were determined by considering the metadata available from Scopus, with the eligible studies meeting the following criteria: (i) be exclusively research articles; (ii) be written in English; and (iii) be published between 2014 and 2024. A limited number of important articles prior to 2014 were also included since they were not previously reviewed thoroughly.

### 2.2. Exclusion Criteria

Reviews, conference papers, book chapters, books, and short surveys, as well as publications focusing on the pharmacokinetic and pharmacodynamic properties of NSAIDs and articles and documents written in languages other than English, were excluded, with the exception of some important review articles that were referred for appropriately quote important aspects on the Introduction and platelet functions sections.

### 2.3. Quality Assessment

To evaluate the articles’ quality and relevance, we first reviewed their titles and abstracts, excluding those unrelated to the topic. Subsequently, the remaining articles were thoroughly read to determine whether they met the predefined inclusion criteria and provided pertinent information for this review.

### 2.4. Selection of Studied NSAID Compounds

NSAIDs encompass various chemical categories and are typically categorized into two main groups: non-selective NSAIDs (nsNSAIDs) and COX-2-selective inhibitors (COXIBs) [24]. Among the most frequently prescribed NSAID compounds are the propionic acid derivatives ibuprofen and naproxen. Another prominent group includes the acetic acid derivatives, from which diclofenac and ketorolac were selected for inclusion. Given their prevalence, we included at least two representative structures from each class. These medications are associated with gastrointestinal (GI) risks, prompting extensive research and discussion in the literature. COXIBs were developed to mitigate the GI side effects of nsNSAIDs [25]. A less commonly used category of NSAIDs is pyrazoles, from which dipyrone was included. Dipyrone, belonging to the pyrazole group, is an analgesic drug widely used for its analgesic, spasmolytic, and antipyretic effects, with fewer gastrointestinal side effects compared to traditional NSAIDs such as ibuprofen and diclofenac [26]. It exhibits inhibitory effects on all COX isoforms (COX1, COX2, and COX3), as well as activation of the opioid and cannabinoid systems. Metamizole’s inhibitory effect on platelet aggregation has been recognized for several years, demonstrated by its dose-dependent inhibition of in vitro platelet aggregation induced by ADP, collagen, epinephrine, and arachidonic acid (AA) [27]. We included representatives from each of these categories.

The category of COXIBs include celecoxib, deracoxib, firocoxib, lumiracoxib, cimicoxib, etoricoxib, lefucoxib, parecoxib, valdecoxib, and robenacoxib [28,29]. Notably, rofecoxib, valdecoxib, and lumiracoxib were withdrawn from the market due to an increased risk of cardiovascular events and mortality [29,30]. Additionally, deracoxib, firocoxib, robenacoxib, and cimicoxib are exclusively used in veterinary medicine, while celecoxib (Celebrex) and etoricoxib (Arcoxia) are administered to humans [28]. Despite belonging to the NSAID class, COXIBs exert their pharmaceutical action by selectively inhibiting COX-2, while some seem to not affect platelet aggregation [20]. Since the efficacy of COXIBs has recently been thoroughly reviewed [28,29], the category of COXIBs was excluded from the present study, which is focused on the interaction and efficacy of the traditional NSAIDs on platelet function and reactivity.

### 2.5. Intended Audience

The findings of this study are targeted towards academic and industrial scientists in the general fields of drugs, pharmaceutics, medicine and pharmaceutical chemistry, biochemistry biology, molecular biology, and chemistry, as well as towards healthcare professionals, and policymakers. The research offers insights into the potential multifaceted role(s) of NSAIDs on platelet function and activation in the presence of absence of anti-platelet therapy.

## 3. Thrombo-Inflammatory Pathways of Platelet Activation as Targets for Drug Action

**Figure 1:**
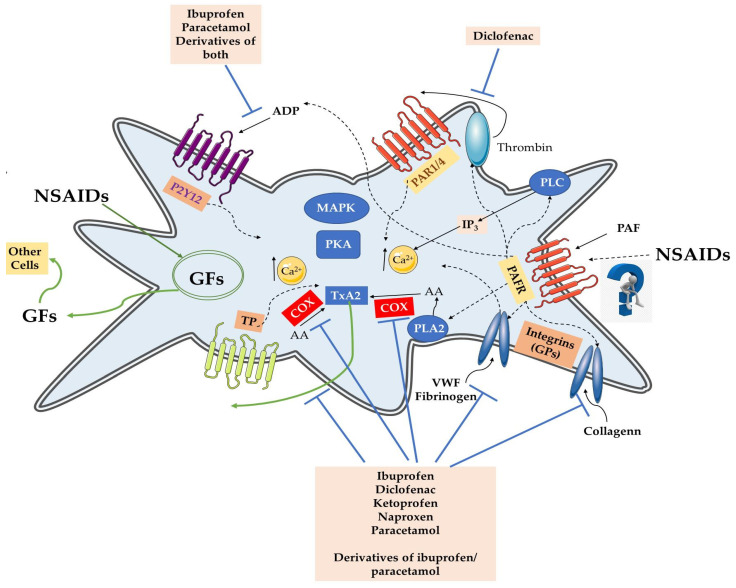
The thrombo-inflammatory signaling of platelet activation and the modulatory effects of each and all NSAIDs on each platelet agonist and its associated receptors. ADP activates the P2Ys receptors’ associated signaling; AA and COX produce TxA2 that activates TP receptors’ signaling; vWF, fibrinogen and Collagen activate GPs (integrins) associated signaling; Thrombin activates PARs receptors’ signaling; PAF activates PAFR signaling that affects all the other platelet agonists’ signaling (i.e., activation of PLC for IP3 production, activation of kinases, activation of PLA2 for production of AA, etc.). Activation of each platelet agonist-receptor signaling results in processes through activation of intracellular signaling by kinases (MAPK, PKA, PKC, PIP3K, etc.), IP3 and [Ca^+2^i], AA, GFs, etc., all of which result in activation and aggregation of platelets. Each NSAID and some of their derivatives were found to modulate several of these thrombo-inflammatory signaling, apart from the PAF/PAFR pathways for which limited information exists. NSAIDs also affect the release of several agents and factors from platelet granules, such as GFs. Abbreviations: NSAID(s) = non-steroidal anti-inflammatory drug(s); AA = arachidonic acid; ADP = adenosine 5′ diphosphate; TP = Thromboxane Prostanoidreceptors; P2Ys = metabotropic P2 receptors, such as the Gq-protein-coupled P2Y1 and the Gi-protein-coupled P2Y12 receptor; PARs = protease-activated receptors (mostly PAR1 and PAR4); TxA2 = thromboxane A2; TxB2 = thromboxane B2; COXs = cyclooxygenases; GPs = glycoproteins as receptors (integrins), such as the integrin αIIbβ3 (glycoprotein GPIIbIIIa), integrin α2β1 (glycoprotein GPIa-IIa) and glycoprotein VI (GPVI); PAF platelet-activating factor; PAFR = receptor of PAF; vWF = von Willebrand factor; GFs = growth factors (i.e., GFsNGF = nerve growth factor; BDNF = brain-derived neurotrophic factor); PLA2 = phospholipase A2; PLC = phospholipase C.

Cell surface receptors play a crucial role in facilitating signal transduction processes. Platelets, versatile blood cells, utilize various receptors to carry out their functions in blood vessels. These functions involve three main types of signals: inhibitory, activation, and negative feedback [31]. The activation and aggregation of platelets are implicated in several physiological and pathological situations [3,4,5,6]. Mediators of thrombosis and inflammation implicated in such procedures and associated disorders have been characterized as well-established and emerging platelet agonists since they can activate platelets by binding to specific receptors for their actions that are located in the membranes of platelets. Several such platelet agonists exist, which affect platelet action through several signaling pathways related to the receptor they bind and depending on the initial stimuli (Figure 1).

Subsequently, creating the next generation of antiplatelet therapy presents the task of striking a delicate balance between the agent’s antithrombotic effectiveness and the risk of bleeding [1]. Hence, investigating the effects of drugs on well-established mediators and their receptors, as well as against novel options and neglected so far receptors holds potential, as they introduce new mechanisms and contribute to diverse pathways or phases of platelet adhesion, activation, or aggregation.

Classic examples of such well-established platelet agonists and their receptors in platelet membranes that pose as drug targets are released from membrane phospholipids arachidonic acid (AA) upon the action of phospholipase A2 that is metabolized in TxA2 into platelets and activates the Thromboxane Prostanoid (TP) receptor [6,11]. Another important pathway is that of the adenosine diphosphate (ADP), the pathways of which also belong to the main drug targets [1,32,33,34,35]. Although ADP is commonly viewed as a mild aggregator, it plays a vital role in hemostasis by acting as a critical cofactor in platelet activation, both in laboratory settings and within the body. Stored in dense granules, ADP is discharged when platelets are stimulated, amplifying activation levels to achieve comprehensive cellular responses. ADP can autonomously activate platelets by binding to two metabotropic P2 receptors: the Gq-protein-coupled P2Y1 and the Gi-protein-coupled P2Y12 receptors. The activation of these receptors by ADP induces an inhibition of adenylyl cyclase (AC) and thus decreases cyclic guanosine and adenosine 3′,5′-monophosphate (cGMP and cAMP, respectively) synthesis that are powerful platelet inhibitors as they affect the glycoprotein (GP) IIbIIIa (GPIIbIIIa), also called integrin αIIbβ3, and maintain it in its inactive form.

Following coagulation activation and/or thrombo-inflammatory induction, the generated by platelets thrombin cleaves its receptors on the platelet surface, i.e., the protease-activated receptors (mostly PAR1 and PAR4), resulting in their activation. The thrombin-related pathway is also considered one of the most well established thrombotic induced platelet activation and aggregation, as dysregulated PAR1 activity is extensively linked to disease pathogenesis and is implicated in several conditions, such as tumor metastasis, inflammatory disorders, and cardiovascular disease, making it another important drug target [5,36].

Moreover, activation of each or some or all of these TP, GPs, P2Ys, and PARs receptors, subsequently affects their associated intracellular signaling, which results in several cellular responses, including increase of the cytosolic Ca^2+^ levels and induction of a conformational change of αIIbβ3 (GPIIbIIIa) towards its active form on the platelet surface that links its substrates, mainly fibrinogen and the von Willebrand factor (vWF), resulting in platelet aggregation [1,6]. The interaction of such CPs integrins with aggregation induced mediators like vWF and fibrinogen is also another site of interest for drug targeting.

Another thrombo-inflammatory indicator of injury is collagen, the signaling of which is also a promising drug target. When released from damaged cells (i.e., endothelial cells) collagen can directly induce platelet activation and aggregation through integrin α2β1 (glycoprotein GPIa-IIa) and glycoprotein VI (GPVI), but also indirectly through glycoprotein Ib-V-IX transmembrane protein complex found on the surface of platelets, which binds to the A1 domain of vWF that is immobilized on collagen upon vascular injury, mediating the attraction and binding of activated platelets to the injured site [31,37,38,39,40,41]. There, the binding of collagen to GPVI induces a conformational change in the platelets shape, rapid calcium influx through the generation of inositol-1,4,5-triphosphate (IP_3_) from the activation of phospholipase Cγ2 (PLCγ2)and phosphoinositide-3 kinase (PI3K), and the production and release of other secondary platelet agonists (i.e., thromboxanes among others) from the platelet granules (degranulation). PI3Kβ, plays a key role downstream of both collagen/GP and ADP/P2Y_12_ binding, making it a possible choice for drug development too [36,42,43,44]. These procedures further stimulate a rapid response to autocrine and paracrine platelet activation, amplifying and sustaining the initial response and resulting in the recruitment of more platelets from circulation and through further activation of integrin αIIbβ3, which subsequently binds fibrinogen and vWF, resulting in platelet aggregation to form a growing hemostatic plug. Fibrinogen facilitates the bridging of glycoprotein IIb/IIIa integrins between platelets and thus maintains the stability of the thrombus, while GPVI is also a receptor for polymerized fibrin and fibrinogen facilitating the amplification of thrombin generation and the recruitment of platelets to clots.

Moreover, thrombo-inflammatory mediators like the platelet-activating factor (PAF) and its lipid-like PAF-analogues (i.e., oxidized polar lipids), which are released by several activated cells upon inflammatory stimuli, including the peroxisomes of platelets, or by oxidative stress conditions, rapidly activate platelets when binding on a specific receptor for PAF (PAFR), inducing platelet aggregation as well as firm adhesion between leukocytes, platelets, and the vascular endothelium [3,4,5,6]. For example, PAF and leukotriene B4 (LTB4), derived from activated platelets, leukocytes or endothelium, but also produced thrombin due to PAF and LTB4 induced pathways, can propagate the activation of platelets and the subsequent activation and adhesion of leucocytes through the interplay of adhesion proteins, chemokines, and their receptors, which are classic steps of atherosclerotic plaque formation. The PAF/PAFR was initially neglected for several years, but recently it has resurfaced as a hotspot for drug targeting since the dysregulation of the PAF/PAFR signaling is implicated in several thrombo-inflammatory manifestations and associated pathological conditions.

Overall, recent outcomes have indicated that all these pathways of platelet activation and aggregation do not act independently, instead, an interplay between several of these pathways and mediators/agonists exists depending on the cause of activation.

## 4. Nonsteroidal Anti-Inflammatory and Analgesic Drugs Exhibiting Pleiotropic Antiplatelet Properties

Concerning chemical structures, NSAIDs can be categorized into four main groups: diaryl-substituted pyrazoles/furanones, sulphonamides, carboxamides/oxicams, and carboxylic acids [45]. These medications inhibit the COX enzymes, which play a crucial role in inflammatory eicosanoid production. The two primary COX isoenzymes, COX1 and COX2 (prostaglandin G/H synthase 1 and 2), are glycosylated, bifunctional, membrane-bound enzymes primarily located in the endoplasmic reticulum that catalyze the conversion of AA to prostaglandin G2 (PGG2) and PGG2 to prostaglandin H2 (PGH2) by peroxidase (POX) activity. Distributed across various tissues in the body, these enzymes impact hemostasis via prostanoid modulation [14], while their activities, levels, and expression have also been characterized as important inflammatory biomarkers since they are implicated in several inflammatory processes and thus serve as important targets for anti-inflammatory approaches, including NSAIDs. NSAIDs, broadly classified into non-selective and selective NSAIDs, encompass a range of medications that apart from their main anti-inflammatory roles also possess the potential to influence platelet function by inhibiting several pathways of platelet activation and aggregation (Figure 1) [20]. Selective COX2 inhibitors, known as COXIBs, selectively inhibit COX2, while nonselective NSAIDs inhibit both isoenzymes [46]. NSAIDs with antiplatelet properties act by permanently suppressing COX1 and COX2 action [46,47], thereby inhibiting thromboxane A2 (TXA2) synthesis, a compound that induces platelet aggregation [47]. An “ideal antiplatelet” agent should effectively block platelet thrombogenesis through various pathways to prevent “platelet resistance” without compromising hemostasis or wound healing [46]. Nevertheless, the effects of NSAIDs on the antiplatelet function of aspirin upon COX-1 can increase the risk of atherothrombotic events as the antiplatelet and cardio-protective effects of aspirin may be suppressed when NSAIDs are co-administered [47]. Therefore, NSAIDs offering both anti-inflammatory/analgesic activities and antiplatelet effects hold potential for treating chronic inflammatory conditions [46].

Aspirin stands out as the predominant antiplatelet therapy for CVD [48]. Initially utilized as an anti-inflammatory agent, aspirin was discovered in 1956 to protect against heart attacks [7]. Over time, it became the most commonly prescribed antiplatelet therapy for primary prevention [49]. The antiplatelet effects of aspirin are associated with the irreversible acetylation of the platelet COX-1, which impedes arachidonic acid’s entry into the active site and subsequently inhibits TxA2 generation and TxA2-induced platelet aggregation, whereas ibuprofen’s action is reversible, merely obstructing the access of arachidonic acid on its COX-1 binding site and thus and providing a transient antiplatelet effect [7,48]. In vivo, aspirin undergoes rapid deacetylation to produce salicylic acid, a metabolite of lower potency that binds to COX in a reversible manner [20]. At the recommended by the European Society of Cardiology (ESC) therapeutic low doses, ranging from 75 to 100 mg per day, aspirin demonstrates a 100–170-fold greater efficacy against the COX-1 associated pathways compared to its effects on COX-2 in platelets [7]. TxA2 is highly unstable and rapidly hydrolyzed to the physiologically stable inactive metabolite, TxB2, and thus, instead of measuring platelet COX-1 activity, serum thromboxane TxB2 measurement is widely used as a surrogate, and also for indicating TxA2 generation and COX-1 specific effects of antiplatelet agents like aspirin [48]. By measuring TxB2 it has been found that several OTC NSAIDs used by patients for pain relief like naproxen for example may potentially pharmacodynamically interact with such a low-dose recommended aspirin prescription for CVD prevention, depending on the timing of administration of each drug [48]. Concomitant use of such reversible NSAIDs COX-1 inhibitors like ibuprofen and naproxen, decrease the antiplatelet actions of aspirin, while non-selective NSAIDs increase the risk of both bleeding and thrombotic events when co-administered with aspirin. This interaction is not found with selective COX-2 inhibitors (‘coxibs’), but these drugs nonetheless increase the risk of thrombotic complications [50]. Thus, apart from the individual effects each NSAID might have on platelets, it is also worth evaluating their potential interactions with well-prescribed clinical practice antiplatelet therapies not only for CVD management and/or prevention [49] but also in the setting of surgery management [51] where they usually are useful medication, with multiple beneficial effects [52].

However, some agonists such as thrombin and PAF can activate platelets in a COX-independent manner. For example, many bacteria induce platelet aggregation in an aspirin-sensitive manner; however, bacteria such as *S. pneumoniae*, which interacts with TLR-2, can activate platelets in an aspirin-independent manner. Thus, it is possible that during several inflammation-related pathologies, and especially during persistent infections, including the SARS-COV-2 viral infection [53], platelets are activated independently of aspirin action, and this may be the reason for the increased mortality rates during COVID-19 independently from the NSAIDs prescription. In these cases, better anti-platelet agents are needed that act through other pathways too.

Taking that into account, it is notable that apart from the classic interactions of NSAIDs with the COX-mediated thrombo-inflammatory activations and with other drugs involved in these pathways like aspirin, NSAIDs have also shown antiplatelet properties by affecting several other pathways of platelet activation and aggregation, such as those of thrombin-, collagen-, AA- and ADP-induced activation of platelets through their specific receptors, both in vitro/ex vitro and in vivo. Thus, it is also worth examining the prospective implications and applications of such an antiplatelet potential of NSAIDs through their effects in each of these pathways involved in platelet activation and aggregation, especially in pathological conditions where these thrombo-inflammatory manifestations take place.

### 4.1. Ibuprofen Effects on Platelets

Ibuprofen stands as the third most widely used drug globally, with an annual consumption of around 200 tons [54]. It is commonly prescribed for conditions such as arthritis, pain relief, and fever management [55] since apart from its anti-inflammatory activities it has also exhibited antipyretic properties and several other pleiotropic benefits. For example, ibuprofen exhibits well-defined antiplatelet effects [20]. Several studies have indicated that ibuprofen attenuates platelet inhibition (Table 1). In vitro, ibuprofen has been found to inhibit ADP-induced platelet aggregation and prostaglandin synthesis and to stimulate the production of NO at micromolar concentrations [56], while it can also dose-dependently inhibit aggregation of platelets induced by other platelet agonists, including AA, epinephrine, and collagen too [57].

For instance, Martini et al. [58] have also demonstrated a robust inhibition of the AA-induced platelet aggregation by ibuprofen at all doses tested in in vitro studies, in platelets from both human and pig blood samples. Inhibition of collagen-induced platelet aggregation of the same potency was also observed in human blood by the same concentrations of ibuprofen, but at ibuprofen doses of four times the standard dosage in pigs’ blood, respectively, suggesting that human blood was more sensitive to ibuprofen antiplatelet actions. Also, ibuprofen did not affect partial thromboplastin time (PTT) or coagulation profiles at these concentrations, but indeed compromised coagulation at higher doses (i.e., 16 times higher than recommended). Moreover, it is worth noting that in cases where potentiation of the platelet activation effect of one platelet agonist due to the presence of another one, as the observed synergistic effect of the co-presence of AA with either 5-hydroxytryptamine (5-HT) or ADP on human platelet aggregation, such enhanced platelet aggregation was successfully inhibited by different cyclooxygenase (COX) inhibitors, including ibuprofen, with half maximal inhibitory effect (IC50) values of micromolar concentrations [59].

Nevertheless, prior administration of ibuprofen nullified the suppressive impact of aspirin on collagen-induced platelet aggregation but did not impact the antithrombotic effectiveness of ASP6537, a highly selective COX-1 inhibitor with a superior ability to aspirin for normalizing TXA2/PGI2 balance, exerting antithrombotic effect without ulcerogenic side-effects [60].

Aspirin and similar compounds suppress COX-mediated platelet activation and aggregation. However, these effects are absent when NSAIDs are present in the study group. Moreover, NSAIDs can hinder the release of bioactive compounds stored in alpha granules, such as growth factors and platelet factor 4, resulting in significant impairment of platelet function [61]. The dual AA-induced platelet aggregation and TxB2 generation serve as pharmacologically targeted platelet function tests for NSAIDs [62]. However, in a separate study [61], ibuprofen markedly influenced the inhibition of aggregation by aspirin, indicating that ibuprofen significantly interfered with aspirin’s platelet inhibitory action. Moreover, both ibuprofen and naproxen hinder aspirin’s access to its COX1-binding site, consequently reducing the extent of platelet thromboxane inhibition achievable by aspirin [14]. Chinatsu Sakata et al. supported this discovery by demonstrating in their research that the antiplatelet efficacy of aspirin notably diminished when guinea pigs were pre-exposed to ibuprofen [60]. In preventive measures against neurovascular stent thrombosis, it is established that initiating dual antiplatelet therapy in the acute phase (typically lasting between 1 and 6 months), followed by maintenance with a single agent, effectively prevents restenosis or ischemic events. Using two antiplatelet agents with distinct mechanisms ensures robust inhibition of platelet aggregation: aspirin continuously inhibits platelet aggregation throughout their lifespan, while clopidogrel achieves thrombosis reduction by irreversibly and non-competitively inhibiting the P2Y12 receptor of the ADP-pathway.

In an in vivo study, 41 patients who underwent neurovascular stent placement and subsequently underwent ventriculoperitoneal shunt (VPS) surgical treatment allowing drainage of excess cerebrospinal fluid (CSF) from the ventricle into the abdomen, while on dual antiplatelet therapy, were divided into two groups; in the first group dual therapy was stopped and substituted with ibuprofen 0.6 g twice daily for five days, while in the second group dual therapy was maintained during surgery and assessment of risk factors for hemorrhagic complications was conducted for both groups. No observed ischemic complications in either group, while hemorrhagic complications were significantly more prevalent in the maintained dual therapy group in contrast to the dual therapy substituted by ibuprofen group, suggesting that ibuprofen can also be utilized as bridging therapy, which entails temporarily halting dual antiplatelet therapy for a five-day duration, Ensuring adequate time has passed since the last intake of ibuprofen minimizes the risk of hemorrhage, as any drug-induced platelet dysfunction can be effectively managed [63]. Moreover, even though platelet dysfunction is a concern for postoperative pain control with NSAIDs, still, administration of ibuprofen to patients undergoing supratentorial brain surgery led to pain relief and patient satisfaction comparable to morphine and paracetamol, without affecting the patients’ blood platelet and clotting functions, as shown by the PFA-100 (platelet function analyzer) system, which is a platelet function analyzer designed to measure primary platelet-dependent homeostasis [64]. This platelet function analyzer (PFA-100) is used to assess platelet activation under high shear stress and stimulation by collagen and ADP or epinephrine.

Thus, even though ibuprofen carries certain well-known gastrointestinal adverse effects associated with dose and patient population, it still poses a comparatively lower risk of cardiovascular and cerebrovascular adverse effects among NSAIDs. Nonetheless, ibuprofen is one of the most commonly used concomitant medications causing pharmacodynamic interactions and potentially increasing the risk of platelet dysfunction in several cases and/or bleeding in OAPs-treated patients [65]. For example, using the same approach, administration of ibuprofen for a week in healthy individuals completing, PFA-100 scores indicated platelet dysfunction in 63% of participants, which normalized after 24 h due to reversible COX-1 inhibition [66]. Moreover, renal and hepatic adverse effects have also been noted with ibuprofen use, which seem to correlate with dosage, concomitant medications, and patient demographics [67].

On the other hand, novel formulations of ibuprofen with other bioactive compounds like arginine have increased the potency and efficacy compared to the parent ibuprofen sodium-salt molecule, since ibuprofen arginate, for example, was able to retain the in vitro observed key functional effects of NSAIDs against AA-induced platelet aggregation in platelet-rich plasma of healthy donors and colon cancer cell killing in human epithelial colorectal adenocarcinoma cell line, Caco-2, with similar or increased potency compared to ibuprofen sodium, which further suggests the potential of ibuprofen derivatives like ibuprofen arginate as efficacious drugs with the possibility of improved cardiovascular safety [13].

The synthesis and platelet protective nature of novel ibuprofen derivatives has also been reported, not only against thrombocytopenia but also against platelet activation and aggregation [68]. Thrombocytopenia is connected with the pathogenesis of several human diseases, including oxidative stress-associated pathologies and oxidative stress-induced manifestations derived by the administration of other drugs like antibiotics that are usually neglected, but they seem to be the most obvious consequence of elevated rate of platelet apoptosis. Ibuprofen derivatives have been found to possess platelet protective efficacy by blocking oxidative stress-induced platelet apoptosis that can reduce thrombocytopenia and associated human pathologies. More specifically, the most potent antioxidant ibuprofen derivative, dose-dependently mitigated the oxidative stress-induced platelet apoptosis in both platelet-rich plasma and washed platelets. The platelet protective nature of this compound was determined by assessing various apoptotic markers such as ROS generation, cytosolic Ca^2+^ levels, PS externalization, cytochrome C translocation, Caspase activation, mitochondrial membrane depolarization, cytotoxicity, LDH leakage and tyrosine phosphorylation of cytosolic proteins. The same compound has also demonstrated potent platelet aggregation inhibitory properties too, as it was able to dose dependently ameliorate platelet aggregation induced by ADP and Collagen and epinephrine at micromolar concentrations. Therefore, such ibuprofen derivative compounds can be estimated as potential candidates in the treatment regime of pathological disorders associated with platelet activation and/or in platelet apoptosis-related thrombocytopenia manifestations and pathologies.

Moreover, apart from its effects as a platelet agonist, ibuprofen was also able to reduce the release of growth factors from platelets’ stores, such as the amyloid precursor protein and nerve growth factor (NGF) and the brain-derived neurotrophic factor (BDNF) [69]. Such platelet-secreted growth factors are associated with inflammation-related neurodegenerative disorders like depression and Alzheimer’s disease, making thus platelets of special interest that may play a potent role in such pathologies. More specifically, incubation of Sprague-Dawley rat platelets with ibuprofen showed that the spontaneous release of both NGF and BDNF were differentially influenced (reduced), in a time-, dose- and calcium-specific pattern, and thus ibuprofen may also exert neuroprotective properties upon these effects on platelet-derived growth factors of the nervous system.

**Table 1:** Representative studies on the effect of Ibuprofen in platelets.

NSAID Drug (s)	Study Design	Effects on Platelets	Ref
AA and Ibuprofen in blood samples from humans and pigs	Iv vitro In blood samples from 4 pigs. Dose: different dosages (from 1 to 20) of 163 mg/mL ibuprofen were added and platelet aggression was examined 15 min later. In vitro. Blood samples from 6 healthy individuals and 6 pigs. Dose: different dosages (from 1 to 20) of 163 mg/mL ibuprofen were added. Platelet aggression was examined 15 min later.	Ibuprofen inhibited AA-/collagen-induced platelet aggregation, with higher sensitivity in human blood samples in blood samples of both human and pigs (collagen induced platelet aggression was degreased to 10% ± 5% for 20 dosages of ibuprofen at pig blood samples) PT and clot formation time remained unchanged by ibuprofen at recommended (x1) doses, while coagulation was compromised at higher ibuprofen doses (x16), as aPTT exhibited a notable extension	[58,70]
ASP6537, aspirin, clopidogrel and Ibuprofen in a FeCl_3_-induced thrombosis model in guinea pigs	In vivo (Animal model). Guinea pigs were divided into 6 groups. Dose: orally administrated 30 mg/kg Ibuprofen 1 h before been administrated 30 mg/kg ASP6537 or 100 mg/kg aspirin.	Prior administration of ibuprofen nullified the suppressive impact of aspirin on collagen-induced platelet aggregation Ibuprofen was not able to affect the inhibitory activity of ASP6537	[60]
Paracetamol and ibuprofen in patients who underwent neurovascular stent placement and subsequently underwent VPS surgery while on dual antiplatelet therapy	In vivo study (Clinical Trial). Participants were 41 patients who were administrated dual antiplatelet therapy and they were divided into 2 groups. Dose: 0.6 g ibuprofen twice a day First group temporarily halted dual antiplatelet therapy (DAPT) for a five-day duration ibuprofen administration while second group continued their DAPT therapy.	Hemorrhagic complications were significantly more prevalent in the group that the dual antiplatelet therapy was maintained during surgery, in contrast to the group that adopted ibuprofen bridging therapy. No observed ischemic complications in patients of both groups	[63]
Aspirin, Ibuprofen in vitro in hPRP or hWP from blood samples of human healthy volunteers	In vitro study. Blood samples were taken from healthy individuals who stopped taking pharmaceuticals compounds 14 days prior. Platelet rich plasma (hPRP) was mixed with 80 μM ibuprofen, aspirin or salicylic acid for 30 min and 23 °C.	Ibuprohfen inhibited the ADP-induced secondary phase of platelet aggregation (from 21.3 ± 5.6 pmol/mL to 14.8 ± 3.4 pmol/mL). Ibuprofen also stimulated the production of NO, a potent inhibitor of platelet aggregation, in the absence of added ADP in both hPRP and hWP. The latent indicates that this effect was not mediated through plasma proteins. Either a temporary or a lasting inhibition of prostaglandin synthesis by ibuprofen resulted in the synthesis of NO in resting platelets.	[56]
acetylsalicylic acid, dexibuprofen, ibuprofen, or flurbiprofen in whole blood samples of healthy donors	In vitro. Blood samples were from volunteers without medication for 2 weeks. Dose: 0.01 to 100 μΜ of acetylsalicylic acid, dexibuprofen, ibuprofen, or flurbiprofen.	Ibuprofen inhibited ADP-/AA-/Collagen-induced platelet aggregation in a dose-dependent manner (IC_50_ (μΜ): ADP: 36.1 ± 2.4, Collagen: 29.8 ± 1.1, AA: 14.7 ± 1.2) Inhibition of the platelet synthesis of both TxB2 (IC_50_ 101 ± 9.46) and LPS-induced PGE2 (IC_50_ 39.33 ± 2.14), as well as the leukocyte production of both PGF1α (IC_50_ 96.32 ± 4.90) and calcium-induced NO (8.55 ± 0.09) synthesis were observed due to incubation of each cells with increasing concentrations of ibuprofen, and increased nitric oxide production	[57]
Different cyclooxygenase (COX) inhibitors, including ibuprofen against platelet aggregation in human blood samples	In vitro. Blood samples were retrieved from healthy individuals who have not taken any medication for 7 days). 45 mL of platelet reach plasma was diluted to 0.5 mL by adding the pharmaceutical compound.	Recommended concentrations of Ibuprofen inhibited the enhanced aggregation of platelets by the co-induction from two different platelet agonists, AA with either 5-HT or ADP (IC_50_: 18.0 ± 1.8).	[59]
Ibuprofen-arginine (an Ibuprofen arginate derivative)	In vitro study. Participants gave blood samples.	The ibuprofen derivative showed improved inhibition of AA-induced platelet aggregation in platelet-rich plasma of healthy donors and colon cancer cell killing in human epithelial colorectal adenocarcinoma cell line, Caco-2	[13]
Ibuprofen derivative	In vitro study. Participants were healthy individuals from whom blood samples were taken. Dose: from 0 to 100 μΜ of every ibuprofen derivative	Antioxidant properties The ibuprofen derivative with the highest antioxidant properties was also able to dose dependently mitigate the oxidative stress-induced platelet apoptosis in both platelet rich plasma and washed platelets, as assessed by various apoptotic markers such as ROS generation, cytosolic Ca^2+^ levels, PS externalization, cytochrome C translocation, Caspase activation, mitochondrial membrane depolarization, cytotoxicity, LDH leakage and tyrosine phosphorylation of cytosolic proteins The same most antioxidant ibuprofen-derivative compound dose dependently ameliorated platelet aggregation induced by ADP, Collagen and epinephrine	[68]
Ibuprofen	In vitro study. Platelets from Sprague–Dawley rats. Dose: 0.3 μΜ ibuprofen. NGF and BDNF were analyzed after 10 and 6-min.	incubation of Sprague-Dawley rat platelets with ibuprofen showed that the spontaneous release from platelets of both NGF and BDNF (10–15%) neural growth factors, implicated in neurodegenerative disorders, were differentially influenced (reduced), in a time-, dose- and calcium-specific pattern, suggesting neuroprotective properties for ibuprofen	[69]

Abbreviations: NSAID(s) = non-steroidal anti-inflammatory drug(s); AA = arachidonic acid; ADP = adenosine 5′ diphosphate; PT = prothrombin time; aPTT = activated partial thromboplastin time; ASP6537 = a selective cyclooxygenase-1 (COX-1) inhibitor; hPRP = human platelet-rich plasma; hWP = human-washed platelets; NO = nitric oxide; TxB2 = thromboxane B2; LPS = bacterial membrane lipopolysaccharide; PGE2 = prostaglandin E2; PGF1α = 6-keto-prostaglandin F1α; 5-HT = 5-hydroxytryptamine; ROS = reactive oxygen species; PS = phosphatidylserine; LDH = lactate dehydrogenase; NGF = nerve growth factor; BDNF = brain-derived neurotrophic factor.

Interestingly, in other cell models that can substitute platelets for indirectly studying the effects of drugs on platelet function, such as the human erythroid leukemia (HEL) cells, which are used after megakaryocytic differentiation with phorbol 12-myristate 13-acetate as an alternative to platelets, where it was observed that ibuprofen significantly inhibited thrombin-induced increases in intracellular Ca^2+^ concentration ([Ca^2+^]i) mobilization, in a concentration-dependent manner, in micromolar concentrations; an effect that was comparable to that of aspirin’s effect in the same cell model [71]. Furthermore, the interaction effects of the simultaneous combined use of aspirin and ibuprofen on thrombin-induced [Ca^2+^]i mobilization, also showed that when the inhibitory effect of aspirin was higher than that of ibuprofen, the effect of aspirin was reduced, whereas when the inhibitory effect of aspirin was lower than that of ibuprofen, the effect of ibuprofen was reduced. These results show that thrombo-inflammatory mediators that also act as platelet agonists in platelets, can induce several effects in other cells and tissues that also bear the receptors of these mediators in their membranes and the associated signaling pathways, which further suggests that the effects and interactions of the NSAIDs like ibuprofen and aspirin on these cells may differ than what has already been reported for platelets, illustrating the need for expanding such studies in other cell models too.

### 4.2. Diclofenac Effects on Platelets

Diclofenac (2-(2,6-dichloroanilino) phenylacetic acid) is a widely used NSAID [72], which has also shown some antiplatelet potential (Table 2). Diclofenac, available in sodium or potassium forms, is utilized as an analgesic in humans, livestock, and domestic animals for inflammation and pain management [73]. The antiplatelet effects of diclofenac involve several actions, such as inhibiting platelet aggregation and reducing P-selectin expression, which serves as an indicator of platelet activation in vivo. Reduced platelet aggregation has also been observed in platelet-rich plasma (PRP) samples obtained from individuals using diclofenac as the NSAID [61]. A substantial suppression of platelet aggregation was evident in response to AA-induced stimulation of PRP samples from NSAID users in comparison to control subjects that were not administered with diclofenac, while no notable variances were observed in both groups with respect to platelet aggregation induced by stimulation from other platelet agonists, including collagen, ADP, or the selective protease activating receptor 1 (PAR1) agonist peptide, TRAP-6, which activates human platelets via the thrombin receptor signaling pathway.

Moreover, administration of diclofenac in patients after coronary artery bypass grafting (CABG) surgery with the use of cardiopulmonary bypass (CPB), did not interfere with the function of platelets and did not cause increased bleeding, while lower CRP levels were observed that may indicate a reduced inflammatory response after CPB [74]. Therefore, diclofenac could be safe for use in patients undergoing CABG surgery but its value in reducing opioid consumption for such patients seems to need further evaluation.

During the acute phase of cold injury in rats, caused by two hours of exposure to −18 °C, the use of diclofenac sodium along with etoricoxib exhibited a notable decrease in D-dimer and serum fibrinogen levels, accompanied by the restoration of thrombin time to normal levels, suggesting that diclofenac affects these coagulation markers in a way that alleviates the effects of hypothermia [75].

On the other hand, findings from a study conducted by Falcinelli et al. in blood samples of patients preparing for cataract surgery, collected before starting treatment and on the day of surgery after 3 days of eyedrop administration of either diclofenac or indomethacin ophthalmic solution, revealed that AA-induced platelet aggregation studied in light transmission aggregometry (LTA) was notably decreased after the administration of ocular indomethacin but not diclofenac in a concentration range of 0.1–0.2 mM. Moreover, differently than indomethacin, diclofenac treatment did not affect circulating platelet P-selectin expression and did not inhibit AA-induced thromboxane B2 (TxB2) generation. These results show that only indomethacin was able to affect the COX-1 activity which predominates in platelets, but not diclofenac. Taking into account that COX-2 predominates in inflamed tissues like surgically damaged conjunctiva it was suggested that diclofenac eye drops are an efficient NSAID approach for managing anterior chamber inflammation, without affecting platelet activation and aggregation.

Additionally, citrated blood samples from both the indomethacin and diclofenac groups were also assessed in the platelet function analyzer PFA-100^®^, which is designed to analyze platelet function and measure platelet-related primary hemostasis, while it also detects qualitative drug-induced platelet defects, including acetylsalicylic acid (ASA)-induced abnormalities, by using the collagen/epinephrine (CEPI) disposable cartridge [62]. Blood samples were aspirated from the sample cup and passed through the aperture in the coated with platelet agonists CEPI membrane cartridge, which induced platelet adhesion, activation and aggregation leading to rapid occlusion of the aperture and cessation of blood flow, which allowed measurement of the closure time (CT), meaning the time taken for the aperture to close. Notably, the CEPI closure time was extended only in the indomethacin group but not in the diclofenac group, suggesting that diclofenac did not affect platelet function as aspirin does so.

Nevertheless, conflicting data exists about diclofenac’s interaction with aspirin, for individuals relying on aspirin-induced platelet inhibition (Table 2). While the majority of studies indicate that diclofenac does not affect aspirin’s antiplatelet function [76,77], a mixed effect was observed with oral diclofenac when compared to the use of aspirin alone, showing a notable reduction in platelet inhibition at certain time points in the collagen agonist, after aspirin was administered, but there was no significant effect in the AA agonist. On the other hand, topical use of the diclofenac patch did not significantly impact aspirin’s antiplatelet activity in both collagen and arachidonic acid agonist groups and might be a safer option than the oral version [77]. When diclofenac sodium was administered alongside aspirin, the antiplatelet and thus thromboprophylactic effect of aspirin against both ADP and collagen pathways remained unaffected. This combination notably reduced platelet aggregation and TxB2 levels, affirming the safety of using diclofenac sodium with aspirin [76].

**Table 2:** Representative studies on the effect of Diclofenac on platelets.

NSAID Drug (s)	Study Design	Effects on Platelets	Ref
Diclofenac and Dexibuprofen	In vivo in 21 participants (11 patients treated with orthopedic injuries and 10 healthy individuals as control group). Dose: Diclofenac 75 mg or Dexibuprofen 400 mg Diclofenac was administrated twice a day for 3.2 ± 2.1 days.	Diclofenac significantly reduced AA-induced platelet aggregation in hPRP from patients undergoing elective orthopedic surgery while receiving such a NSAID(IC_50_ for ACP was 2.7 ± 3.7, while for control group was 563 ± 61) There were no notable variances observed in platelet aggregation responses to collagen, ADP, or TRAP-6 stimulation when contrasted with the control participants. PRP generated from individuals who have taken such NSAID exhibit notable deficiencies in platelet function. Should such an NSAID administration be necessary, it should be carried out subsequent to blood collection for autologous PRP preparation to avoid potential diminishment of therapeutic efficacy.	[61]
Diclofenac and Aspirin	In vivo (Clinical Trial) in 12 healthy volunteers. Εach participant was randomly classified in 1 of 5 cases of medicines administration, with a cleanse period between treatments. Dose of each category: (a) aspirin 325 mg and after 2 h 50-mg of oral diclofenac potassium, (b) topical diclofenac epolamine 1.3%, (c) diclofenac patch (twice a day) followed by aspirin 325 mg after 21 h, (d) oral diclofenac potassium 50 mg, and (e) aspirin 325 mg. Blood samples were taken every 0, 2, 4, 6, 8, 12, 24, 48, and 96 for each case.	Diclofenac patch in healthy subjects did not hinder the antiplatelet effects of aspirin in both collagen and AA agonists (collagen agonist 95% CI –284.609 to 79.942; AA agonist 95% CI –117.479 to 310.395). Oral diclofenac exhibited varying effects on aspirin-induced inhibition of platelet aggregation (for collagen 95% CI 302.568 to 971.765 while for AA P = 0.973 95% CI –173.506 to 546.756).	[77]
Diclofenac sodium and Aspirin	In vivo (Clinical Trial) in 18 healthy people (segregated into 3 groups). Dose: 150 mg of aspirin once a day and 50 mg of diclofenac sodium 3 times a day for 6 days without the consumption of any other pharmaceutical.	When administering aspirin in conjunction with three daily doses of diclofenac sodium in healthy subjects, the antiplatelet efficacy and thus the thromboprophylactic effect of aspirin remained intact, against both ADP and collagen induced platelet aggregation (minor inhibition of 22.10% and 38,87%, respectively) and in relation to the decrease in the mean TxB2 levels (reduced to 702.99 ± 101.59 pg/mL from 971.11 ± 128.91 pg/mL)	[76]
Diclofenac or Indomethacin	In vivo study (Clinical Trial). 20 patients for cataract surgery participated in a simultaneous randomized trial. Dose: 1 mg/mL Diclofenac or 1 mg/mL Indomethacin for 3 days 4 times a day.	Administration of diclofenac eye-drops in patients preparing for cataract surgery did not result in a significant reduction in AA-induced TxB_2_ generation and platelet aggregation, as well as in circulating platelet P-selectin expression.	[62]
Diclofenac sodium and etoricoxib	In vivo study (Animal model). 41 male rats were exposed to –18 °C for 2 h Dose: 7 mg/kg to diclofenac sodium alleviate the effects of hypothermia	Diclofenac sodium exhibited a notable decrease in D-dimer (from 2454.0 ± 250.3 to 1660.0 ± 293.6) and serum fibrinogen levels (from 306.8 ± 34.4 to 237.1 ± 29.2), accompanied by the restoration of thrombin time to normal levels [75].	

Abbreviations: NSAID(s) = non-steroidal anti-inflammatory drug(s); AA = arachidonic acid; ADP = adenosine 5′ diphosphate;TRAP-6 = peptide fragment that is a selective agonist of the protease activating receptor 1 (PAR1); PRP = platelet-rich plasma; TxB2 = thromboxane B2.

### 4.3. Ketoprofen Effects on Platelets

Ketoprofen (KET) (2-(3-benzoylphenyl) propionic acid) is a widely used NSAID known for its over-the-counter availability and affordability. It is commonly prescribed for alleviating pain in muscles and joints, as well as for treating conditions like arthritis, gout, rheumatoid osteoarthritis, and inflammation in general [78]. Ketoprofen has shown notable antiplatelet effects mostly in animals. More specifically, Ketoprofen treatment in dogs suffering from arthropathies with normal hemostasis profile (*n* = 21) decreased both ADP- and epinephrine-induced platelet aggregation of dogs’ PRP, without influencing the shape change, lag time values and feature of the platelet aggregation curve [79]. On the other hand, the therapeutic doses of ketoprofen in dogs caused a significant increase on the closure time of the PFA-100 testing of their blood, which is a marker of platelet function, platelet activation and platelet aggregation, when using cartridge with a collagen-epinephrin stimulation, suggesting that ketoprofen affects also the hemostatic properties of platelets [80]. Thus, the literature is controversial with regards to hemostasis and NSAIDs like ketoprofen in dogs and this might be explained by different drugs, dosage regimen, animal population, and primary or secondary hemostatic function tests (i.e., buccal mucosal bleeding time, thromboelastography, prothrombin time, activated partial thromboplastin time, etc.) (Table 3).

For example, platelet aggregation time (PAT), was not affected in ketoprofen treated dogs for osteoarthritis [15], ketoprofen administration preoperatively to female dogs undergoing ovariohysterectomy, a significant decrease in collagen-induced platelet aggregation was observed, although buccal mucosal bleeding time did not change [81]. ANSAID-dependent platelet aggregation decrease in ketoprofen-treated dogs when compared with placebo-treated dogs has been observed in other studies too, through inhibition of COX-1 and consequent synthesis of TxA2 [82]. However, it seems from most studies that these effects are not clinically meaningful as bleeding time was rarely affected after NSAID administration. Reduction in ex vivo serum TxB2 concentrations indicated marked inhibition of platelet COX-1 after both oral and intravenously (IV) administration of enantiomers of Ketoprofen to cats at clinically recommended dose rates, has also been reported [83].

Ketoprofen and other NSAIDs have shown reduced production of TxB2 from both platelets and mononuclear cells in humans too [84], due to marked inhibition of platelet COXs. Nevertheless, katoprofen and other propionic acid-derived NSAIDs, have shown notable thrombocytopenia side-effects, and thus they should be used with caution, especially in patients with compromised platelet function. More specifically, Razi, M.T et al. investigated the potential mechanism underlying thrombocytopenia associated with ketoprofen use, identifying as a possible cause the ketoprofen-induced inhibition lactic dehydrogenase (LDH) in platelets, since LDH is a pivotal enzyme for platelet energy metabolism and thus LDH’s functionality is necessary for platelet activation and aggregation. Based on these outcomes it was suggested that patients requiring uncompromised platelet function and are prescribed with ketoprofen should undergo monitoring for platelet count and blood clotting during and just after such an administration [85].

Lucarini et al. confirmed that Hybrid compounds constituted by an Inhibitor of Carbonic Anhydrase (CAI), which is an enzyme implicated in inflammation-related pulmonary fibrosis, connected to a NSAID were found to be promising new anti-inflammatory drugs for the treatment of lung chronic inflammatory diseases, while these hybrid compounds under investigation did not affect the inhibition of platelet aggregation and the TXB2 production in hPRP compared to reference molecules [86].

**Table 3:** Representative studies on the effect of Ketoprofen on platelets.

NSAID Drug(s)	Study Design	Effects on Platelets	Ref
Ketoprofen	In vivo examination in animals. Specifically, 115 dogs (43 healthy, 44 diseased and 21 with arthropathies) Dose administered: carprofen (2–4 mg/body weight two times a day) or ketoprofen (1 mg/body weight one time a day) for at least ten days.	Ketoprofen treatment in dogs suffering from arthropathies with normal hemostasis profile decreased ADP (inhibition 61%)- and epinephrine (inhibition 41%)-induced PRP platelet	[79]
Ketoprofen	In vivo examination in animals (22 healthy dogs). Dose: 11 dogs, ketoprofen (2 mg/kg) and 11 dogs, 0.9% NaCl solution (control). Surgery was performed on the animals after dosing.	Ketoprofen administration preoperatively to female dogs undergoing ovariohysterectomy, resulted in a significant decrease in collagen (inhibition 95% and 80%)-induced platelet aggregation, without any alterations in bleeding time.	[81]
Ketoprofen	In vivo examination in animals. Experiment 1 Animals 6 healthy cats The study lasted 3 periods with 2 weeks between each period. Experiment 2 Animals eight healthy cats. The study lasted 2 periods. Dose: 1–2 mg/kg, ketoprofen No adverse effects were observed in either experiment 1 or experiment 2.	Reduction in ex vivo serum TxB2 concentrations indicated marked inhibition of platelet COX-1 (inhibition 90%) after both oral and intravenously administration of enantiomers of Ketoprofen to cats at clinically recommended dose rates	[83]
Ketoprofen and other NSAIDs	In vitro Three human blood samples and human mononuclear cells from four human blood samples.	Ketoprofen showed reduced production of TxB2 from both platelets (inhibition 99%) and mononuclear cells (inhibition 100%) in human blood samples, due to marked inhibition of platelet COXs.	[84]
Ketoprofen	In vitro Samples humans’ blood. Dose: (250, 500, 750, 1000 and 1500 µg/mL) concertation ketoprofen.	Ketoprofen demonstrated competitive inhibition of LDH activity in human platelets. Ketoprofen, whether used alone or in combination with other treatment protocols, is associated with Thrombocytopenia (a reduction in platelet count), posing a significant risk to patients who depend on normal unimpaired platelet function for their well-being, suggesting monitoring for platelet count and blood clotting during ketoprofen prescription. Maximum LDH activity of 89% was found using 1500 μg/mL of the ketoprofen.	[85]
Ketoprofen ibuprofen, (S)-(+)-naproxen, -(−) and sulindac.	In vitro Platelets and Macrophage cells Dose: 100 nM, 1, 10 and 100 M of each compound.	The hybrid compounds under investigation showed no elevation in the inhibition of platelet aggregation when compared to reference molecules. Furthermore, these compounds were not more potent in attenuating the activity of the COX-1 pathway compared to their original molecules.	[86]

Abbreviations: NSAID(s) = non-steroidal anti-inflammatory drug(s); ADP = adenosine 5′ diphosphate; PRP = platelet-rich plasma; TxB2 = thromboxane B2; COX = cyclooxygenase; LDH = lactate dehydrogenase.

### 4.4. Naproxen Effects on Platelets

Naproxen belongs to a category of medications renowned for their anti-inflammatory, pain-relieving, and fever-reducing properties. It functions by reducing the production of chemicals that cause pain or fever in the body. The timing and sequence of drug administration also affect their inhibitory effects on platelets (Table 4). Naproxen, like ibuprofen, seems to hinder the acetylation of COX-1 by aspirin by competitively binding to COX-1 [20].

Paul A. Gurbel’s research revealed a pharmacodynamic interaction that endured for a minimum of three days after ceasing naproxen intake following ten days of simultaneous usage of low-dose of aspirin and naproxen sodium. On the initial day of concurrent dosing, there was no significant interaction observed, with the singular use of naproxen sodium having no impact on aspirin-induced thromboxane inhibition. The timing of naproxen administration appears pivotal when administered over multiple days, and this interaction could potentially be alleviated by taking immediate-release aspirin at least 30 min before naproxen [48].

Astrid S. Clarke et al. discovered that when administered alone, lanraplenib (GS-9876), aspleen tyrosine kinase inhibitor for autoimmune diseases or naproxen or ibuprofen or aspirin, all were able to inhibit platelet aggregation induced by convulxin and AA in vitro. However, no significant improvement in platelet aggregation inhibition when any of them were mixed with GS-9876 was found across all donors. Although some donors exhibited a ≥50% enhancement in platelet aggregation inhibition when treated with combinations of aspirin and GS-9876 or Naproxen and GS-9876, this effect varied among the combination groups and donors [87].

### 4.5. Metamizole Effect on Platelets

Studies have suggested that dipyrone may mitigate the antiplatelet effects of aspirin, both experimentally and in cohorts of patients hospitalized for exacerbation of coronary artery disease [88,89]. As dipyrone acts as a reversible inhibitor of prostaglandin formation [90], this interaction likely arises from competition with aspirin at the level of platelet cyclooxygenase (COX-1). The effects of metamizole on platelets are summarized in Table 5.

Research by J. Papp et al. confirmed that the antiplatelet effect of metamizole and acetylsalicylic acid did not significantly differ In vitro. These observations suggest a competitive interaction between the two drugs. In vivo experiments demonstrated that intravenously administered metamizole is an effective antiplatelet agent and can serve as a therapeutic alternative when oral acetylsalicylic acid cannot be used [91].

A study by Amin Polzin et al. in 2015 demonstrated that the interaction between aspirin and dipyrone, leading to inadequate antiplatelet effects of aspirin, can be prevented by a strict order of intake. Administering aspirin prior to dipyrone intake ensures sufficient antiplatelet effects of aspirin. Additionally, their data suggests that the mechanism involves a direct pharmacodynamic drug-drug interaction at the level of COX-1 [92].

According to a study by Andrea Schmitz et al., dipyrone may have undesirable effects in addition to rare agranulocytosis and may diminish the antiplatelet effect of aspirin. Specifically, platelet thromboxane synthesis was higher in patients receiving dipyrone plus aspirin compared to controls. In vitro measurements with blood from healthy individuals confirmed that dipyrone significantly reduces the inhibition of platelet thromboxane synthesis by aspirin [93].

**Table 5:** Representative studies on the effects of metamizole on platelets.

NSAID Drug (s)	Study Design	Effects on Platelets	Ref
Metamizole and Aspirin	In vivo (Clinical Trial). A single-blind randomized controlled study with 43 participants diagnosed with coronary artery disease, divided into two groups. Group 1 received metamizole and opioids post-operation, while Group 2 received only opioids. Aspirin was administered prior to the use of metamizole. The dosage regimen consisted of metamizole at 125 mg every 8 h for 6 days, accompanied by a daily dose of 300 mg of aspirin for the same duration.	There was no alteration in platelet activation when metamizole was administered before aspirin. The function of collagen-activated platelets remained unchanged with the use of metamizole. In the first group, after six days, it measured 1776 ± 429 AU min, and in the second group, it was 1225 ± 288.2 AU min.	[94]
Metamizole and aspirin	In vivo (Clinical Trial) in 27 participants with cardiac diseases or pain symptoms who were prescribed both metamizole and aspirin. As a control group, 10 individuals were given aspirin without any other pharmaceutical intervention. The dosage regimen included daily administration of aspirin ranging from 75 to 150 mg for 7 days, along with metamizole at a daily dose ranging from 10 to 20 mg/kg for the same duration.	Metamizole altered platelet inhibition when administered alongside small doses of aspirin. In the control group, arachidonic acid-induced platelet aggregation was activated in only 10% of cases, while in the group prescribed with metamizole, this percentage reached 78%. This finding was further corroborated by the ex vivo study, wherein the control group demonstrated inhibition of platelet aggregation with aspirin alone. However, when aspirin was co-administered with metamizole, inhibition was not achieved (TXB_2_ 14 ± 7).	[93]
Metamizole and aspirin	In vitro in platelets; platelet aggregation was assessed using blood samples from 10 individuals to which metamizole and aspirin were added. The doses administered were 6, 12, and 25 μg/mL of each drug. Additionally, 1 μg/mL of metamizole and 2 μg/mL of aspirin were used to examine potential interactions. In vivo (Clinical Trial) in 20 healthy individuals divided into three groups. The first group received intravenous diluted metamizole sodium, while the second group was orally administered aspirin. The third group received both intravenous metamizole and aspirin. The doses administered were 1 g/mL of diluted metamizole sodium, 250 mg of metamizole, and aspirin at varying concentrations over a 72-h period.	In vitro: Metamizole exhibited antiplatelet properties comparable to aspirin. Both drugs demonstrated antiplatelet effects at high concentrations; however, at smaller doses (6 and 2 μg/mL), aspirin inhibited platelet activation while metamizole did not. In vivo: intravenously administered metamizole achieved platelet aggregation inhibition in a shorter timeframe compared to aspirin (metamizole maximum inhibition time: 4 min, aspirin maximum inhibition time: 7 h). Orally administered metamizole also achieved inhibition after several hours. Co-administration of metamizole and aspirin was not as effective as administration of aspirin alone.	[91]
Metamizole and aspirin contains observations from l study that has not been referenced	In vitro in platelets from blood samples obtained from 7 volunteers. Different concentrations of aspirin and metamizole were used. In vivo (Clinical Trial): Four individuals were divided into 2 groups. Group 1 received aspirin for 3 days followed by metamizole, while Group 2 received only aspirin. After a washout period, each group underwent co-administration of both drugs. The dosage administered was 750 mg/day of metamizole and 100 mg/day of aspirin. In vivo (Clinical Trial): Twelve participants were divided into two groups. The first group received aspirin, followed by metamizole 30 min later, while the second group received metamizole first, followed by aspirin after 30 min. The dosage administered was 100 mg/day of aspirin and 750 mg/day of metamizole for 7 days.	Interactions between metamizole and aspirin can alter platelet inhibition. At lower concentrations, while aspirin was able to deactivate platelet aggregation, metamizole did not exhibit the same effect. Co-administration of both drugs failed to inhibit arachidonic acid (AA)-induced thromboxane (TX) formation. However, when aspirin was administered at higher concentrations (300 and 1000 μΜ), inhibition was achieved. Administering aspirin half hour before metamizole resulted in platelet inhibition. Therefore, both the dosage and the sequence of drug consumption influence their effectiveness.	[95]
Metamizole and aspirin	In vivo study (Clinical Trial) that involved 37 individuals scheduled for thoracic surgery, divided into two groups. The first group had not taken NSAIDs, while the second group was treated with a combination of aspirin and metamizole. Dosage: 100 mg of aspirin.	While metamizole can exhibit antiplatelet effects, its administration alongside aspirin can lead to implications. When metamizole was administered alone, it resulted in inhibition of arachidonic acid (AA)-induced platelet aggregation. The effectiveness of the drug remained consistent regardless of the method of administration within the initial minutes (95% CI from 1.03 to 0.885). Platelet activation induced by thrombin receptor-activating peptide (TRAP) was reduced from 89.6% to 78.7% following metamizole use. However, combined administration of the two pharmaceuticals altered platelet inhibition in 40% of the patients.	[92]
Metamizole and aspirin	In vivo (Clinical Trial), in 36 volunteers that were administered metamizole and aspirin for 7 days, in comparison to a reference group. Dosage: Aspirin at 100 mg once daily. Metamizole was administered either before or after aspirin.	The concurrent administration of aspirin and metamizole may interact with platelet aggregation. When metamizole was administered before aspirin, the high on-treatment platelet reactivity (HTPR) was reduced by 20%. However, when metamizole was given after aspirin, the HTPR increased.	[95]

In the following year, Pfrepper et al. confirmed that metamizole induces a strong inhibitory effect on arachidonic acid (AA)-induced platelet aggregation, detectable for up to 41 h in some patients. While there was a less pronounced effect on collagen-induced platelet aggregation, all other inducers showed no effect. In some hospitalized patients, no aspirin-induced inhibition of platelet aggregation was detectable, irrespective of the sequence of administration [26].

Mirosław Wilczyński et al. evaluated the impact of intravenous metamizole on platelet inhibition by aspirin in patients with coronary artery disease early after on-pump coronary artery bypass grafting (CABG). Intravenous metamizole, preceded by a loading dose of aspirin, did not alter platelet response to aspirin in the postoperative period after CABG [94].

In recent years, Christian Pfrepper et al. aimed to analyze the inhibitory effect of aspirin and metamizole on AA-induced platelet aggregation throughout the day. Co-medication of aspirin and metamizole significantly influences platelet inhibition, with variations observed during the day, potentially leading to high on-treatment platelet reactivity in patients taking aspirin prior to or simultaneously with metamizole [95].

### 4.6. Paracetamol Effects on Platelets

Paracetamol, while not categorized as an NSAID, operates as a reducing cosubstrate at the peroxidase site of cyclooxygenase (COX), resulting in reversible inhibition. Additionally, metabolites of NSAIDs might demonstrate COX inhibitory properties [20].

The impact of paracetamol (acetaminophen) on platelet function has been previously reviewed by Driver et al. [20], but several aspects of its effects on platelets still remain not fully clarified and understood, with studies yielding varied results (Table 6). The majority of the studies have suggested that paracetamol has limited or no antiplatelet effects when compared to aspirin, while some studies have shown that paracetamol possess antiplatelet effects. Another factor that has been studied also is the timing and sequence of NSAID co-administration with paracetamol, which seems to play a crucial role on paracetamol’s effects on platelets, as concurrent NSAID usage can either inhibit or enhance platelet activation, contingent upon the specific NSAID utilized [96,97].

## 5. NSAIDs as Emerging Contaminants

NSAIDs like ibuprofen in platelets and other cells, it should also not be neglected that the high prescription rate of ibuprofen is followed by its detection in several water basins. As these medications are not completely absorbed by the body, they are excreted through urine.

NSAIDs are characterized as weak organic acids containing a carboxylate group [98]. Their acid dissociation constants (pKa) range from 4.00 to 4.91, while their octanol-water partition coefficients (Kow) range from 1.10 to 3.97. This suggests that NSAIDs predominantly exist as dissolved neutral species under typical environmental conditions [99]. Their high water solubility and polar nature pose challenges for their removal efficiency in WWTPs [100].

Consequently, there is a possibility of their entry into water bodies, and they may not be effectively eliminated by conventional wastewater treatment methods [101], which has led to the their characterization as emerging contaminants (ECs) or contaminants of emerging concern (CECs), raising public health concerns due to their persistence in the environment [102]. NSAIDs often detected at trace levels in water bodies, including drinking water supplies. Emerging contaminants are substances found in global drinking water sources in minimal amounts, with uncertain risks to human health and aquatic ecosystems. Despite their historical presence in drinking water, recent advancements in analytical technology have facilitated their detection [103,104]. It has been observed that NSAIDs enter drinking water primarily through surface runoff and wastewater treatment plant (WWTP) effluents [105]. Effluents from WWTPs, which can impact receiving water bodies, serve as the primary source of pharmaceuticals in surface waters potentially used for drinking water. Additionally, pharmaceutical discharge into groundwater from sources like pipelines and sewage leaks may contribute to NSAID contamination in drinking water [106]. NSAIDs, among the most commonly used pharmaceuticals globally and listed among the top 10 persistent pollutants, have posed challenges due to limited understanding of their occurrence, distribution, and eco-toxicological effects over the past decade [107]. NSAIDs have been detected in various water sources [108].

For example, concentrations of ibuprofen have been found to reach up to 1400 μg/L in surface waters, up to 1670 μg/L in wastewater, and up to 6000 μg/kg in sediments [109]. In addition, diclofenac, is also classified as an emerging contaminant [73]. Global consumption estimates suggest approximately 940 tons of diclofenac are used annually, making it one of the most detected pharmaceuticals in aqueous effluents. According to reported data, diclofenac levels in municipal wastewater can reach up to 7.1 µg·L^−1^ [72]. It should also be noted that widespread utilization of ketoprofen has resulted in its detection in water bodies, with concentrations ranging up to 210 μg/L [101]. While concentrations in natural waters typically remain below 1 μg/L, higher levels are often observed in rivers that receive untreated wastewater discharge, emphasizing the need for effective wastewater treatment. These environmental and public health concerns pose as a limitation of their use and should not be neglected too, suggesting that following its increased prescription trend there is also a need for developing novel and improved green methodologies for its removal from water sources.

A range of wastewater treatment technologies has been explored to address this issue, including conventional methods, as well as advanced techniques such as membrane filtration and adsorption [110]. However, adsorption has garnered attention as a cost-effective alternative to more traditional options for large-scale application [111]. Thus, further research should prioritize enhancing wastewater treatment methods with cost-effective, efficient, and sustainable technologies, addressing current adsorbents’ limitations like cost and regeneration. It is crucial to evaluate the long-term effects of NSAIDs on ecosystems and human health to establish safe concentrations in aquatic environments. Tackling challenges in adsorption processes, including capacity and cost, is essential.

## 6. Limitations and Future Perspectives

The efficacy of NSAIDs in antiplatelet therapy has been acknowledged, but the available data need further evaluation The study primarily examines the antiplatelet properties of representative NSAIDs, namely ibuprofen, naproxen, diclofenac, ketoprofen, and paracetamol, within the timeframe of 2014–2024. All these NSAIDs showed considerable and multifaceted effects on platelet activation, aggregation, and overall functions, as well as on platelet viability in several conditions. However, the effects of these drugs on platelet functions still have not yet been fully evaluated. For example, these NSAIDs have not yet been studied against other important thrombo-inflammatory signaling that hugely affect platelet function in several pathologies. More specifically, the effects of these NSAIDs on PAF-associated inflammatory signaling have not yet been reported.

Thus, the observed effects of these drugs on specific platelet agonists and their receptors in platelets can be extended in other mediators, and several other cells/cell-models and tissues that also possess such thrombo-inflammatory signaling machinery. Moreover, cell models of other cells imitating platelets can facilitate the study of the effects of these drugs on platelet function without affecting platelets. Interestingly, novel derivatives of several of these drugs, as was observed in the case of ibuprofen derivatives, have also exhibited promising enhanced outcomes against platelet-associated thrombo-inflammatory manifestations, while the inhibition of the release from platelets of several growth factors related to neurodegenerative disorders may pose as alternative neuroprotective approaches.

The potential of dual antiplatelet therapy is also discussed, but still the study lacks a comprehensive assessment of its favorable benefit/risk profile. Moreover, there was no assessment of outcomes stratified by NSAID dose due to limitations in statistical power. The comprehensive evidence concerning specific NSAID risks, dose-response associations, and the risk over time primarily stems from observational studies. However, these studies are restricted by unaccounted confounding variables and lack the ability to conclusively establish causation. Also, despite extensive research on well-established antiplatelet agents’ effects, such as aspirin’s effects on platelet function, understanding their interaction(s) with other NSAIDs and related drugs like paracetamol remains limited. Research is needed on concurrent NSAID usage, the influence of NSAID metabolites on platelet function and viability, and their effects on platelet quality during storage, transfusion and setting of several surgeries.

## 7. Conclusions

Within the present study, the antiplatelet potential of commonly administered NSAIDs like ibuprofen, diclofenac, naproxen, ketoprofen, and paracetamol was thoroughly reviewed along with mechanisms of actions against different pathways of platelet activation and aggregation. This article highlights the main outcomes of in vitro and in vivo studies related to such an additional health-promoting effect for these anti-inflammatory and analgesic agents, along with the caution needed with respect to their dose-efficacy, safety, and interactions upon co-administration with classic antiplatelet therapy with well-established platelet agonists. The limitations, environmental impact, and future perspectives on the use of NSAIDs were also outlined.

## Figures and Tables

**Table 4:** Representative studies on the effects of naproxen on platelets.

NSAID Drug (s)	Study Design	Effects on Platelets	Ref
Low-dose aspirin and over-the-counter naproxen sodium	In vivo (Clinical Trial) in 117 humans. The study consisted of 3 periods: Period one lasts 6 days (1–6), 81 mg ASA, second period lasts 10 days (7–16) concurrent treatment, 220 mg naproxen and 81 mg ASA and third period lasts 3 days (17–19), 81 mg ASA. Adverse effects such as gastrointestinal, nervous system, reproductive system and breast, etc.	By administering immediate-release naproxen once or twice daily concurrently with a low-dose regimen of immediate-release aspirin, in healthy subjects, no pharmacodynamic interaction was observed within the initial 24 h of such a concurrent therapy. A pharmacodynaminmic interaction was observed after ten days, which persisted even after discontinuing naproxen. Inhibition 94.86% and 95%	[48]
GS-9876, naproxen, ibuprofen, and aspirin	In vivo and In vitro Animals monkeys Dose: 5, 15, and 45 mg/kg Humans (Samples blood) Dose: 15–50 mg one time a day for seven days.	When PRP from heathy monkeys and humans abstaining from NSAID use for 1 week, were pretreated separately in GS-9876, naproxen, ibuprofen, and aspirin, an inhibition of platelet aggregation induced by a combination of convulxin and AA was observed. In the combinations of Naproxen + GS-9876, four out of nine donors displayed higher than 50% enhancement in the inhibition of platelet aggregation. Nonetheless, this response was inconsistent across all combination groups and donors.	[87].

Abbreviations: NSAID(s) = non-steroidal anti-inflammatory drug(s); AA = arachidonic acid; PRP = platelet-rich plasma; GS-9876 = lanraplenib, a second-generation spleen tyrosine kinase (SYK)inhibitor.

**Table 6:** Representative studies on the effects of paracetamol (acetaminophen) on platelets.

NSAID Drug (s)	Study Design	Effects on Platelets	Ref
Paracetamol (Acetaminophen) and meloxicam	In vitro Platelets from human blood samples from six healthy humans. Dose: 214 μg/mL the standard dose, 1T), 4T, 8T, 10T, 12T, 16T, and 20T acetaminophen. Similar dosages were used for the meloxicam. The Food and Drug Administration (FDA)-approved standard dose for acetaminophen is 15 mg/kg every 6h. The FDA-approved standard dose for meloxicam is 0.2 mg/kg	Notable suppression of AA-induced platelet aggregation in PAWB of healthy subjects was noted with acetaminophen and/or meloxicam, even at their standard therapeutic doses. Similarly, collagen-induced platelet aggregation was inhibited by acetaminophen (inhibition 72 +/− 10%of control) or meloxicam (inhibition 72+/− 5% of control), starting from doses as low as 1 or 4 of the recommended doses. Both acetaminophen and meloxicam, whether administered individually or together, demonstrated the ability to hinder platelet aggregation, even at typical doses.	[96]
2-acetamidophenol (a positional isomer of paracetamol)	In vitro in human platelets Dose: 1, 5, 50, and 100 µM concentrations of 2-acetamidophenol. Adverse effects: with minimal or no risk of gastric ulcer.	2-acetamidophenol exhibited notable activity against AA (inhibition 93.8 +/− 2.9% for dose 1 μM)-induced platelet aggregation in PRP of healthy subjects 2-acetamidophenol exhibited lower sensitivity against the ADP (inhibition 52+/− 1.4% for dose 50 μM)-induced platelet aggregation, an effect which however was of greater potency and efficacy than that of aspirin’s against ADP Additionally, 2-acetamidophenol showed significantly higher potency than paracetamol.	[97]

Abbreviations: NSAID(s) = non-steroidal anti-inflammatory drug(s); AA = arachidonic acid; ADP = adenosine 5′ diphosphate; PRP = platelet-rich plasma; PAWB = platelet-rich whole blood.

## Data Availability

All data analyzed during this study are included in this published article.
